# Molecular Imprinted Based Quartz Crystal Microbalance Nanosensors for Mercury Detection

**DOI:** 10.1002/gch2.201800071

**Published:** 2018-11-27

**Authors:** Sabina Hüseynli, Duygu Çimen, Nilay Bereli, Adil Denizli

**Affiliations:** ^1^ Department of Chemistry Hacettepe University Beytepe Ankara 06800 Turkey

**Keywords:** mercury(II), molecular imprinting, quartz crystal microbalance, wastewater

## Abstract

Mercury(II) ions are emerging as a result of more human activity, especially coal‐fired power plants, industrial processes, waste incineration plants, and mining. The mercury found in different forms after spreading around diffuses the nature of other living things. Although the damage to health is not yet clear, it is obvious that it is the cause of many diseases. This work detects the problem of mercury(II) ions, one of the active pollutants in wastewater. For this purpose, it is possible to detect the smallest amount of mercury(II) ions by means of the mercury(II) ions suppressed quartz crystal microbalance nanosensor developed. Zinc(II) and cadmium(II) ions are chosen as competitor elements. Developed nanosensor technology is known as the ideal method in the laboratory environment to detect mercury(II) ions from wastewater because of its low cost and precise result orientation. The range of linearity and the limit of detection are measured as 0.25 × 10^−9^–50 × 10^−9^ m. The detection limit is found to be 0.21 × 10^−9^ m. The mercury(II) ions imprinted nanosensors prepared according to the obtained experimental findings show high selectivity and sensitivity to detect mercury(II) ions from wastewater.

## 1 Introduction

As a result of rapid population growth, and industrialization, wastewater has exceeded than that nature can cope with, and the receiving environment faces the risk of pollution.^1^, ^2^, ^3^, ^4^, ^5^ The need to purify the wastewaters from hazardous metals has arisen in order to prevent this situation that could affect the ecological balance.^6^, ^7^, ^8^ Contaminants present in wastewater can be dissolved in either water or solid matter.^9^, ^10^ Mercury is one of the persistent pollutants in wastewater, and when it reaches surface waters or soils, microorganisms may transform it into methyl mercury, a substance that is rapidly absorbed by most bodies and is known to cause nerve damage.^11^, ^12^, ^13^, ^14^ Acid surface waters can contain significant amounts of mercury.^15^ When the pH is between five and seven, the concentration of mercury in the water increases. Because of this, there is a severe environmental and public health problem, and it has become necessary to detect it in the aqueous environment.^16^, ^17^, ^18^, ^19^

The monitoring of Hg(II) ions levels in water (drinking, sea, lake, etc.) is very important in terms of waste management, environmental analysis, toxicology, water safety, and water quality.^4^ The limit of acceptable Hg(II) ions concentration in water is 10 × 10^−9^ m according to the U.S. Environmental Protection Agency (EPA).^20^ The detection and measurement of Hg(II) ions from water are based on spectroscopic techniques such as atomic absorption, emission, and mass spectroscopies.^21^ Although these methods offer high selectivity and sensitivity, they are costly, nonportable, time‐consuming, and they often require multi‐stage steps and professional operators, and they are also labor‐intensive. Therefore, low cost, simple, rapid, and portable methods for Hg(II) ions detection are highly desired. In recent years, quartz crystal microbalance (QCM) nanosensor has become a glamorous alternative for the analysis of mercury with higher detection limits. Herein, we have combined the advantages of the QCM nanosensor and molecular imprinting polymer. Molecular imprinting technique is based on molecular recognition and provides specific recognition cavities in polymer matrices with memory of the template molecules.^22^ QCM is a selective, cost effective, simple, and high resolution mass sensing technique that detects the mass change of quartz crystal surface by measuring the change in resonance frequency in real time. Several methods are available for the increase of the sensitivity of QCM nanosensor. QCM has been commonly applied in environmental assays, biology, life sciences, analytical chemistry, and pharmaceutical sciences.^23^ Molecular imprinting method–based QCM nanosensors with high selectivity and sensitivity are developed for real‐time detection of Hg(II) ions.

In the study, *N*‐methacryloyl‐(l)‐cysteine (MAC) and Hg(II) ions were co‐complexed, and then Hg(II) ions were imprinted into the pHEMAC film by the most effective and low cost method of molecular imprinting.^24^, ^25^ The molecularly imprinted (MIP) nanosensors are a rapidly developing subject and have given rise to many important developments at the same time.^26^, ^27^, ^28^, ^29^ In this experiment, characterization studies were performed with Fourier‐transform infrared spectroscopy (FTIR), contact angle, atomic force microscopy (AFM), and ellipsometer measurements of both Hg(II) ions imprinted and non‐imprinted pHEMAC nanofilms. At the same time, selectivity and kinetic studies, intraday and interday measurements were performed. Precise work has been done to determine the reproducibility of the recommended method. For repeatability, four samples from the same concentration were prepared and measured. This day‐to‐day study was carried out at three different times a day at the same concentration. The same procedure was observed for three different days. The Hg(II) ions imprinted pHEMAC nanofilm we obtain is very attractive due to its high biocompatibility, selectivity, surface modification, reusability, and relatively low cost.^30^, ^31^

## 2 Results and Discussion

### 2.1 Preparation and Characterization of Hg(II) Ions Imprinted QCM Nanosensors

**Figure 1:**
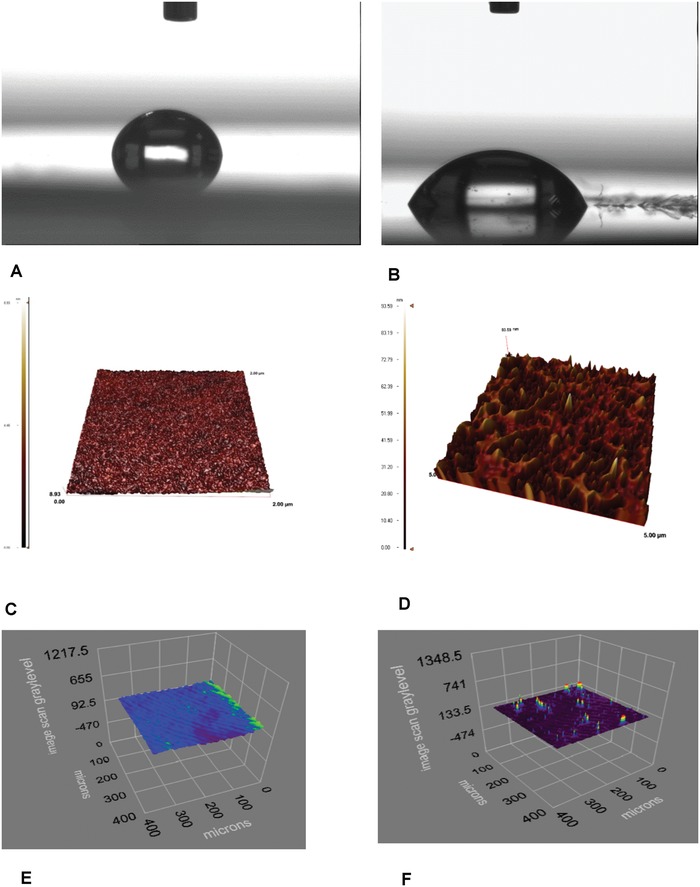
Contact angle: A) bare gold surface, B) Hg(II) ion imprinted pHEMAC QCM chip, AFM studies: C) bare gold surface, D) Hg(II) ion imprinted pHEMAC QCM chip, ellipsometry images, E) bare gold surface and F) Hg(II) ion imprinted pHEMAC.

Surface morphology of the Hg(II) ions imprinted pHEMAC nanosensors were characterized with FTIR, contact angle, AFM, and ellipsometry dimensions and performed to calculate the thickness of polymeric film onto the gold surface of QCM nanosensor chips. FTIR spectrum of MAC has the characteristic stretching vibration amide I adsorption bands at 1606 and 1387 cm^−1^. For the characteristic determination of pHEMAC polymer, the characteristic strong –SH stretching vibration bands at 3021 cm^−1^ slips to the higher frequency field at 2910 cm^−1^ as a result of decreasing the electron density of sulfhydryl group of MAC monomer. The FTIR spectrum of MAC has showed the characteristic –SH absorbance peak at 3021 cm^−1^ shifted to 2910 cm^−1^ due to MAC incorporation into the HEMA monomer and was confirmed by carbonyl stretching bands at 1713 cm^−1^. Hydrophilicity of nanofilm was determined by contact angle measurements. The contact angle of the unmodified QCM nanosensor decreased from 81.4° to 67.2° when the Hg(II) ions imprinted pHEMAC nanofilm was attached onto the modified gold surface (**Figure** **1**A,B). Decrease in the contact angle shows the increased hydrophilic property of nanosensor chip surface. The surface morphologies of Hg(II) ions imprinted pHEMAC nanosensors were investigated with AFM measurements (Figure 1C,D). The surface depths of Hg(II) ions imprinted QCM nanosensors were determined with AFM as 8.03 and 93.59 nm, respectively. AFM images indicate clearly that a polymeric film was synthesized on the nanosensor surfaces. The ellipsometric thicknesses were measured as 92.5 ± 0.8 nm for gold QCM surface (Figure 1E) and 113 ± 0.7 nm for Hg(II) ions imprinted QCM nanosensor (Figure 1F). For the results of ellipsometry, we can say the chip surfaces are homogeneous. MAC monomer contains carboxylic acid group and has hydrophilic structure. These results are consistent with results of AFM.

### 2.2 Kinetic Analyses with Hg(II) Ions Imprinted QCM Nanosensors

**Figure 2:**
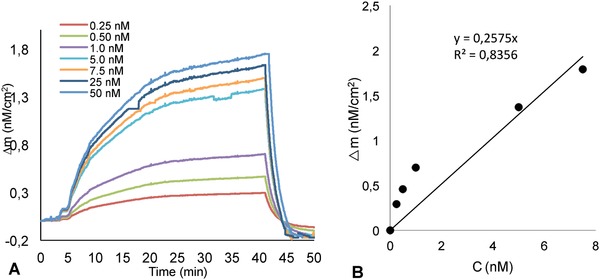
Real‐time responses and linear regions of QCM nanosensors aqueous solutions of Hg(II) ions at different concentrations (A) and standard calibration curve (B).

The detection of Hg(II) ions from an aqueous solution was performed using Hg(II) ions imprinted and non‐imprinted pHEMAC nanosensors. First, Hg(II) ions imprinted pHEMAC nanosensor was equilibrated with 1% HNO_3_ solution. After, a series of various concentration solutions of Hg(II) ions ranging from 0.25 × 10^−9^ to 50.0 × 10^−9^ m were applied to QCM system in **Figure** **2**A. As seen in the figure, the increase in Hg(II) ions concentration caused an enhancement in QCM nanosensor response.

The plateau was reached after 35 min and 1.0% HNO_3_ solution was injected onto the QCM nanosensor for washing of unbounding molecules. Initially, the nanosensor's response increases linearly, and then it reaches its plateau at a relatively high concentration of Hg(II) ions (10.0 ng mL^−1^) as saturation is the accessible imprinted voids. The whole cycle containing adsorption, desorption, and regeneration was completed in about 50 min. Figure 2B shows the linear range of Hg(II) ions imprinted pHEMAC nanosensor (0.25 × 10^−9^–7.5 × 10^−9^ m).

The data were obtained from this concentration range, which was used to determine the limit of detection (LOD) and limit of quantitation (LOQ) values of the Hg(II) ions imprinted pHEMAC nanosensor.

Limit of detection was calculated by the parity

(1) LOD=3.3S/m

Limit of quantification was conjectured by the parity

(2) LOQ=10S/m

where *S* is the standard deviation of the intercept and *m* is the slope of the regression line.^32^, ^33^, ^34^ LOD and LOQ values were calculated to be 0.21 × 10^−9^ and 0.73 × 10^−9^ m, respectively. A summary of the different detection methods for Hg(II) ions is given in **Table** **1**.

**Table 1:** The collation of various Hg(II) ion detection methods

Technology	Linear range	LOD	Refs.
SPR	0 × 10^−6^–50 × 10^−6^ m	1 × 10^−6^ m	35
Electrochemical sensor	0.5–150 µg L^−1^	0.2 µg L^−1^	36
Electrochemical square wave voltammetry	1.0 × 10^−8^ mol L^−1^–1.0 × 10^−5^ mol L^−1^	5.8 × 10^−9 ^mol L^−1^	37
Screen‐printed electrode	0.5–10 mg L^−1^	0.2 mg L^−1^	38
Fluorescent sensor	0.1 × 10^−6^–1 × 10^−6^ m	6.8 × 10^−9^ m	39
QCM nanosensor	0.25 × 10^−9^–50 × 10^−9^ m	0.21 × 10^−9^ m	This study

To evaluate the applicability of the QCM nanosensor, water sample (sampled from Beytepe, Ankara, Turkey) was spiked with Hg(II) ions and tested in the QCM nanosensor. To further demonstrate the applicability of our QCM nanosensor in practical applications, we performed recovery experiments using spiked water sample with 1.0 × 10^−9^ m of Hg(II) ions. We observed an average recovery value of 92%, indicating that the QCM nanosensor can be used for the detection of Hg(II) ions in water sample even at concentrations below the allowed Hg(II) ions concentration (10 × 10^−9^ m) defined by the U.S. Environmental Protection Agency. High recovery percentages even at very low Hg(II) ions concentration and low standard deviation in the experiments indicate the high accuracy of our QCM nanosensor.

Kinetic analysis of Hg(II) ions imprinted and non‐imprinted pHEMAC nanosensors was performed in aqueous solution in real time. Freundlich, Langmuir, and Langmuir–Freundlich adsorption isotherm models were calculated by using experimental results. The adsorption parameters are given **Table** **2**.

**Table 2:** Kinetic and isotherm parameters

Association kinetic analysis		Equilibrium analysis (Scathard)			
*k*_a_ (ng mL^−1^ s^−1^)	0.0094	∆*m*_max_ (ng cm^−2^)	1.20		
*k*_d_ (1 s^−1^)	0.008	*K*_A_ (ng mL^−1^)	1.19		
*K*_A_ (ng mL^−1^)	1.175	*K*_D_ (mL ng^−1^)	0.83		
*K*_D_ (mL ng^−1^)	0.85	*R* ^2^	0.986		
*R* ^2^	0.971				
Langmuir		Freundlich		Langmuir–Freundlich	
∆*m*_max_ (ng cm^−2^)	1.66	∆*m*_max_ (ng cm^−2^)	1.49	∆*m*_max_ (ng cm^−2^)	0.20
*K*_D_ (mL ng^−1^)	1.25	1/*n*	0.61	1/*n*	0.61
*K*_A_ (ng mL^−1^)	0.80	*R* ^2^	0.999	*K*_D_ (mL ng^−1^)	0.71
*R* ^2^	0.994			*K*_A_ (ng mL^−1^)	1.39
				*R* ^2^	0.928

**Figure 3:**
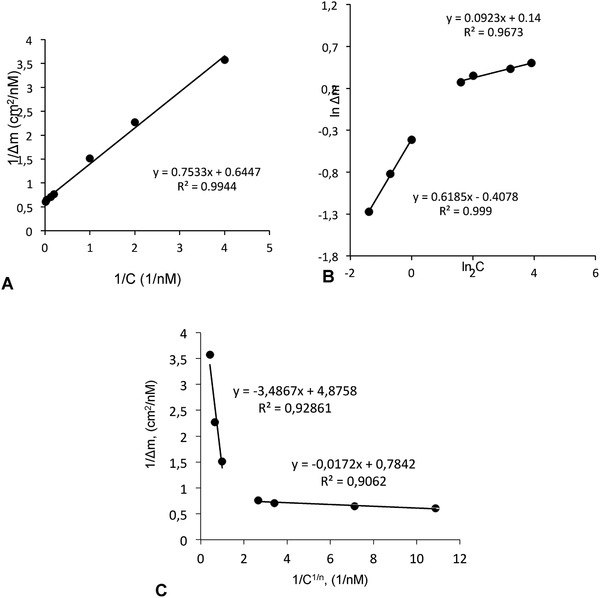
Langmuir (A), Freundlich (B), and Langmuir–Freundlich (C) adsorption models.

According to the obtained data, this results are in good agreement with the Langmuir model (*R* ^2^: 0.999), indicating that the prepared binding sites for Hg(II) ions on the nanosensor surface are monolayer, co‐energy, homogeneously distributed, and minimal lateral interaction (**Figure** **3**A,B). The Δ*m*_max_ value calculated from Langmuir model is very close to the value obtained experimentally (Δ*m*_max_: 1.66 ng mL^−1^). The Scatchard plot analysis indicated that the polymer binds a single molecule to each binding site (*R* ^2^: 0.986), which confirms the good fit of the Langmuir model.

### 2.3 Selectivity of Hg(II) Ions Imprinted QCM Nanosensors

**Figure 4:**
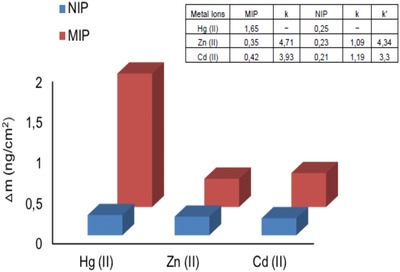
Comparison of selectivity of QCM nanosensors; the nanosensor response of Hg(II), Zn(II), and Cd(II) ions on Hg(II) ion‐imprinted and non‐imprinted pHEMAC QCM nanosensors.

The selectivity of Hg(II) ions imprinted pHEMAC nanosensor was examined using Cd(II) and Zn(II) ions. Selective recognition of Hg(II) ions with Hg(II) ions imprinted and non‐imprinted pHEMAC nanosensors was examined with 25.0 × 10^−9^ m of each Hg(II), Cd(II), and Zn(II) ions solutions. The selectivity coefficients (*k*) and relative selectivity coefficients (*k*′) valuation are dedicated in **Figure** **4**. Hg(II) ions imprinted QCM nanosensors were 10.48 and 9.35 times more picky for Hg(II) ions whence Cd(II) and Zn(II) ions, seriatim. Fastening capacities of Hg(II) ions imprinted and non‐imprinted pHEMAC nanosensor were compared. As seen in Figure 4, fastening capacity of Hg(II) ions imprinted pHEMAC nanosensor is higher than non‐imprinted pHEMAC nanosensor. It shows that the Hg(II) ions imprinted pHEMAC nanosensor recognizes Hg(II) ions with good selectivity because of the Hg(II) ions imprinting methods that chemical recognition cavity and create shape.

### 2.4 Reproducibility and Stability

**Figure 5:**
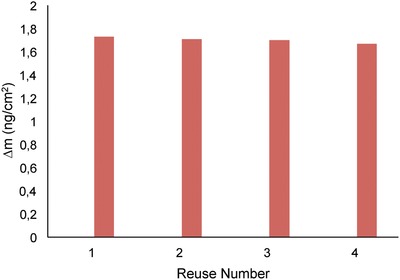
Reproducibility of Hg(II) ion‐imprinted pHEMAC nanosensor (Hg(II) ion concentration: 50 nM).

**Figure 6:**
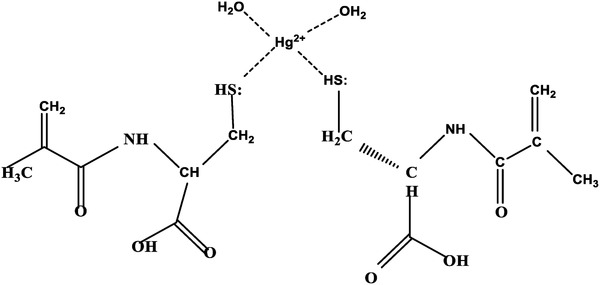
The molecular formula of MAC‐Hg(II) ion complex monomer.

The equilibration–adsorption–regeneration cycles were repeated for four times using aqueous Hg(II) ions solution with concentration of 50 × 10^−9^ m in **Figure** **5**. As can be seen from Figure 6, Hg(II) ions imprinted pHEMAC nanosensor has displayed reproducible mass shift during the cycles and Hg(II) ions imprinted pHEMAC nanosensor shows that there is no decrease in adsorption capacity during four cycles. Four samples were prepared and measured at the same concentration to perform the repeatability study.

For the intraday study, the Hg(II) ions solution was prepared at the same concentration. The sample was prepared at three different times of the day. Intraday sensitivity was also determined by the same procedure. Interday application was observed for three different days. The result was recorded as %RSD. Average %RSD was 1.119. The consequences of the studies are shown in **Tables** **3** and **4**.

**Table 3:** Precision results showing repeatability

Concentration (nm)	Δ*m*	Statistical analysis
50	1.70	
50	1.67	Mean 1.7
50	1.71	SD 0.023
50	1.73	% RSD 1.079

**Table 4:** Intraday (A) and Interday (B) precision

Concentration (nm) (A)	Δ*m*1	Δ*m*2	Δ*m*3	Average% RSD
50	1.68	1.69	1.68	
50	1.69	1.69	1.66	
50	1.67	1.67	1.64	
50	1.65	1.65	1.63	
% RSD	1.021	1.143	1.341	1.062
Concentration (nm) (B)	% RSD			Average% RSD
	Day 1	Day 2	Day 3	
50	1.097	1.120	1.141	1.119

## 3 Conclusion

As a result, we have developed a new method of quartz crystal microbalance nanosensor to detect Hg(II) ions from wastewater. In this method, we first converted the MAC and Hg(II) ions into the precomplex and then modified the nanosensor chips to obtain pHEMAC‐Hg(II) ions and pHEMAC polymers. Characterization analyses were made with high precision, and the obtained polymers were compared with previous studies. The range of linearity and the limit of detection were measured as 0.25–50 × 10^−9^ m. The detection limit was found 0.21 × 10^−9^ m. Compared to other methods in literature, our method is known to be as cheap and fast as it is sensitive. Moreover, it is also favored in terms of time and timing. Rapid increase in population, urbanization, and industrialization, excessive and unconscious use of fossil fuels such as coal, natural gas, and oil, and increase in consumption per capita are the main factors accelerating the pollution and deterioration process. This study contributes to the detection of heavy metal ions from wastewaters resulting from environmental pollution.

## 4 Experimental Section

*Materials*: Mercury (II) nitrate (Hg(NO_3_)_2_), zinc (II) nitrate (Zn(NO_3_)_2_), cadmium (II) nitrate (Cd(NO_3_)_2_), allyl mercaptane (CH_2_CHCH_2_SH), 2‐hydroxyethylmethacrylate (HEMA), ethylene dimethacrylate (EDMA), *N*,*N*′‐azobisisobutyronitrile (AIBN), and sodium chloride (NaCl) were supplied from Sigma‐Aldrich. All other chemicals were used as received and were bought from Merck A.G. (Darmstadt, Germany). Ultrapure deionized water (resistivity ≥ 18 MΩ cm) was used pending the trials, and it was cleansed by Barnstead (Dubuque, IA, USA) ROpure LP reverse osmosis unit.

*Surface Modification of the QCM Chips—Modification of QCM Chips with Allyl Mercaptane*: The gold surfaces of QCM chips were used to clean with 10 mL of acidic piranha solution (3:1 concentrated H_2_SO_4_:H_2_O_2_, v/v) for 5 min. At that time, QCM chips were cleaned with ethyl alcohol and were desiccated in vacuum oven (200 mmHg, 40 °C). After this process, the gold‐coated surface of the QCM chips was connected with allyl mercaptane (CH_2_CHCH_2_SH). After modification procedure allyl groups were cleaned from the gold‐plated QCM chip surface with ethyl alcohol and desiccated with nitrogen gas. At the completion of surface modification QCM chips were rinsed with ethyl alcohol to remove unbounded allyl mercaptan and desiccated with nitrogen gas.

*Surface Modification of the QCM Chips—Preparation of Hg(II) Ions Imprinted and Non‐Imprinted pHEMAC Nanosensors*: For the preparation of Hg(II) ion imprinted and non‐imprinted pHEMAC QCM nanosensors, MAC:Hg(II) ions pre‐complex as template molecule Hg(II) ions was prepared by using Hg(II) ions and MAC monomer (**Figure** **6**).

The stoichiometric molar ratio of MAC:Hg(II) ions pre‐complex was determined by preparing in different molar ratio as 1:1, 2:1, 3:1. The effect of the increasing ratio of the functional monomer (MAC) upon the complex formation was examined with 1240 mini SHIMADZU UV‐1601 model spectrophotometer (Tokyo, Japan). The absorbance of MAC:Hg(II) ions was restrained in range of 200–700 nm wavelength. The absorbance of MAC:Hg(II) ions increases with increasing ratio of MAC. The maximum absorbance value was observed in 1:2 ratio of MAC:Hg (II) ions. Δ*m* and imprinting factor (IF) values were obtained with Hg(II) ion imprinted (MIP) and non‐imprinted (NIP) pHEMAC nanosensors prepared with MAC:Hg(II) ions containing polymerization mixtures at different mole ratios. The imprinting factor for Hg(II) ion imprinted and non‐imprinted pHEMAC nanosensor was calculated as follows

(3) $$\mathrm{The}\;\;\mathrm{imprinting}\;\;\mathrm{factor}\;\;\left(\mathrm{IF}\right):\Delta m_{\left(\mathrm{MIP}\right)}/\Delta m_{\left(\mathrm{NIP}\right)}$$

First, Hg(II) ions (750.0 µL) and MAC (10.0 µL) MAC:Hg(II) ions (1:2) were stirred at room temperature for 2 h. Then 4.0 mg of AIBN as initiator was dissolved in 3.76 µL of EGDMA and 1.21 µL of HEMA. The prepared MAC:Hg(II) ions complex was mixed with this solution. Thereafter, 5.0 µL was pulled of the stock monomer solution with pipette and modified with allylmercaptan on QCM chip using the spin coating method. Polymerization was carried out by means of UV‐irradiation (100 W, 365 nm) for 45 min at room temperature under a nitrogen atmosphere. The QCM chip was washed four times with ethanol and desiccated in a vacuum oven. 0.05 m EDTA and 0.05 m HCl solutions were utilized as the desorption agent to detect Hg(II) ions from the QCM nanosensor. The non‐imprinted pHEMAC nanosensor was prepared by applying the same procedure without Hg(II) ions.

*Surface Modification of the QCM Chips—Instrumentation*: Characterization studies of Hg(II) ion imprinted and non‐imprinted pHEMAC nanosensors were completed with FTIR, contact angle, AFM, and ellipsometer measurements. The characterization of pHEMAC and Hg(II) ion imprinted pHEMAC nanosensor was performed using FTIR spectrophotometer (Thermo Fisher Scientific, Nicolet iS10, Waltham, MA, USA) in the wavenumber range of 700–4000 cm^−1^. For the contact angle of the chips surface, KRUSS DSA100 (Hamburg, Germany) apparatus was used and one drop was mesured by sessile drop method. The different regions of the QCM chip surfaces were selected. This was repeated five times and images were recorded. The thickneses measurements of QCM polymeric films were performed using an ellipsometry (Nanofilm EP3, Germany) at a wavelength of 532 nm with an angle of incidence of 62°. Quantifications were repeated three times in six different locations on the nanosensor surface and the consequences are recorded based on the average of these values. Surface topographies of QCM chips were investigated with AFM (Nanomagnetics Instruments, Oxford, UK) in tapping mode. Kinetic studies of Hg(II) ion imprinted pHEMAC QCM nanosensors were carried out with QCM system (INFICON Acquires Maxtek Inc., New York, USA).

*Surface Modification of the QCM Chips—Real Time Detection and Kinetic Studies with Hg(II) Ions Imprinted pHEMAC Nanosensors*: The real‐time detection of Hg(II) ions from an aqueous solution was performed with Hg(II) ion imprinted pHEMAC nanosensors. First, Hg(II) ion imprinted QCM nanosensor was equalized with 25  × 10^−3^ m acetate buffer, pH 5.0 buffer. After, aqueous solutions of Hg(II) ions in a concentration range between 0.25 × 10^−3^ and 50.0 × 10^−3^ m were applied to QCM system. The whole cycle containing adsorption, desorption and regeneration was completed about 40 min. In order to detect Hg(II) ions from Hg(II) ion imprinted pHEMAC nanosensor surface, 0.05 m EDTA and 0.05 m HCl solutions were used as a desorption solution. After the each desorption cycle, the surface of pHEMAC nanosensors was washed with deionized water and 0.1 m phosphate buffer, pH 7.4, respectively. For the selectivity of Hg(II) ion imprinted pHEMAC nanosensor was used Cd(II) and Zn(II) ions. Finally, Hg(II) ions determination from the water sample was performed with Hg(II) ion imprinted pHEMAC nanosensor in order to show the reliability of the designed QCM nanosensor. Hg(II) ions solution 1.00 × 10^−9^ m was spiked in water sample. Thereafter, the prepared water sample was applied to QCM system.

## Conflict of Interest

The authors declare no conflict of interest.
